# Does Finishing at Pasture Influence the Colour of Muscle from Suckler Bulls and Can Colour Be Used to Authenticate Their Pre-Slaughter Diet?

**DOI:** 10.3390/foods11152281

**Published:** 2022-07-30

**Authors:** Aidan P. Moloney, Edward G. O’Riordan, Mark McGee, Brigitte Picard, Frank J. Monahan, Lara Moran, Raquel Cama-Moncunill

**Affiliations:** 1 Teagasc, Animal & Grassland Research and Innovation Centre, Grange, Dunsany, Co., C15PW93 Meath, Ireland; edward.oriordan@teagasc.ie (E.G.O.); mark.mcgee@teagasc.ie (M.M.); 2 Institut National de Recherche pour l’Agriculture, l’Alimentation et l’Environnement (INRAE), VetAgro Sup, Unité Mixte de Recherche sur les Herbivores (UMR Herbivores), Université Clermont Auvergne, F-63122 Saint-Genès-Champanelle, France; brigitte.picard@inrae.fr; 3 School of Agriculture and Food Science, University College Dublin, D04V1W8 Dublin, Ireland; frank.monahan@ucd.ie (F.J.M.); raquel.cama-moncunill@ucd.ie (R.C.-M.); 4 Teagasc Food Research Centre, Ashtown, D15KN3K Dublin, Ireland; lara.moran@ehu.es; 5 Lactiker Research Group, Department of Pharmacy and Food Science, University of the Basque Country (UPV/EHU), 01006 Vitoria-Gasteiz, Spain

**Keywords:** bulls, grazing, longissimus muscle, colour, discrimination, muscle fibres

## Abstract

The primary objective of this study was to compare the colour of muscle from bulls finished at pasture or indoors on a high concentrate diet. The ancillary objectives were to identify possible explanations for any differences in the colour observed and the potential of muscle colour to discriminate between bull beef from different production systems. Growth, longissimus muscle colour, fibre type composition and metabolic profile were measured in late-maturing breed sired suckler bulls slaughtered at 19 months of age after 199 days at pasture (G0), 100 days indoors after 98 days at pasture (G0AL) and indoors for 199 days (AL). When compared to bulls finished indoors and offered a high concentrate ration, the carcass weight of G0 bulls was lower, their carcasses were leaner, and their longissimus muscle was similar in lightness but less red and had a lower glycolytic metabolism. The temperature at which the longissimus muscle reached pH 6.0 was lower (19.7 °C) for G0 than for G0AL (29.9 °C) and AL (31.6 °C), which did not differ. Co-variate adjustment for this variable removed the differences in redness. Adjusting the chill settings appears to be a practical strategy for abattoirs to minimise early post-mortem differences in muscle colour between lighter grass-fed and heavier concentrate-fed carcasses. The preliminary results demonstrate the potential of both L*, a*, b* values and the visible reflectance spectra of muscle to discriminate between grass- finished and concentrate-finished bull beef, but further refinement and validation of the models is required.

## 1. Introduction

Colour is an important influence on the decision of a consumer to purchase a particular cut of beef [1]. Animals that are finished on pasture often have darker meat than similar animals finished indoors on a high concentrate diet, but this is not always the case [2]. Changes in colour due to the production system may therefore influence the perception of the consumer with regard to the quality of the meat. Most research on the effects of grass finishing on muscle colour relates to steers and heifers. From the perspective of the beef producer, bulls rather than steers are attractive because of their superior growth rate and feed conversion characteristics [3]. Traditionally, bulls have been finished indoors on high concentrate diets to allow them to achieve their genetic potential for growth but also recognising that the hormonal characteristics of bulls make them more difficult to manage in a pastoral situation. Consequently, there is a paucity of data on the colour of muscle from grass-finished bulls. We have previously shown that after a short period at pasture prior to slaughter, when measured at the commercial carcass cutting point (fifth/sixth rib interface), bulls had darker (i.e., a lower L, lightness, value) meat that was less red (i.e., a lower a value), than similar bulls finished indoors [4]. The colour of the longissimus muscle (LT) posterior to the 10/11th rib interface, the commercially valuable striploin cut, was not reported. It is not known whether the differences in colour at this position in the carcass compared to bulls reared indoors on a high concentrate ration would persist over the course of a long grazing season facilitated by the temperate climate in Western Europe. This was the first objective of this study with the ancillary objective of identifying possible explanations for any differences in colour observed.

The provenance of beef is also of increasing importance to the consumer, in particular whether beef comes from animals that are finished on pasture or had pasture in their diet. “Grass-fed” and “grass-based” are among the labels used on beef products to reflect this interest of beef consumers and to capture a premium in the marketplace. Several approaches to protect these labels have been examined. For example, beef from cattle finished on pasture or concentrates could be distinguished based on their fatty acid composition, stable isotope ratios or near infrared or Raman spectroscopic spectra [5]. These approaches require destructive chemical analysis and/or expensive equipment. In contrast, when measuring muscle colour parameters (lightness, redness and yellowness) using a portable or bench-top spectrophotometer, the visible reflectance spectrum can be simultaneously obtained.

Huang et al. [6] reported that the visible reflectance spectrum of perirenal fat could discriminate between lambs that consumed grazed grass and a concentrate diet indoors (95% correct classification) but did not evaluate this approach for muscle. Prieto et al. [7] concluded that the visible part of the visible–NIRS spectrum (350–750 nm) was unsuccessful in discriminating between dark and normal beef muscle, but no data were provided. More recently, Barragan et al. [8] reported 70% and 66% correct classification of muscle from cattle finished on a barley- or corn-based ration, respectively, using the visible part of the visible–NIRS spectrum. Accordingly, our second objective was to determine the potential of both the colour parameters and the reflectance spectrum of muscle to discriminate between beef from bulls that grazed grass only or concentrates before slaughter.

## 2. Materials and Methods

This study was carried out under license from the Irish Government Department of Health and Children and with the approval of Teagasc, the Agricultural and Food Development Authority (Teagasc Animal Ethics Committee, approval #78/2014). All procedures used complied with national and EU regulations concerning experimentation on farm animals.

### 2.1. Animals, Management and Post-Slaughter Measurements and Sampling

Forty-five spring-born late-maturing breed sired bulls, of approximately 8 months of age, were sourced from commercial beef suckler herds, based on breed availability in October. During the indoor winter period, animals were offered grass silage ad libitum, supplemented with 1.6 kg of concentrate dry matter daily (862 g rolled barley, 60 g soya bean meal, 50 g sugar cane molasses and 28 g of a mineral and vitamin mixture per kg, crude protein = 130 g/kg dry matter, metabolisable energy = 12.4 MJ/kg dry matter). At the end of the winter, the bulls were weighed on two consecutive days and blocked on a combination of sire breed and weight and assigned at random within blocks of 3 animals (*n* = 15/treatment; 9 Charolais and 6 Limousin) to one of three treatments: (1) grazed grass for 199 d (G0); (2) grazed grass for 98 d, then housed in a concrete slatted floor shed (in pens of 5 animals per treatment) and offered the above concentrates plus grass silage ad libitum for 101 d (G0AL); (3) housed as above and offered concentrates plus grass silage ad libitum for 199 days. The pen dimensions were approximately 4.5 m × 3.5 m. The feeding space, 4.5 m, was adequate to allow all bulls to access the ration at the same time. Bulls rotationally grazed *Lolium perenne*-dominant swards to a target post-grazing sward height of 4.5 cm within separate treatment farmlets as previously described [9]. Rotations were managed so that there were no bulls in the paddock immediately adjacent. The average space allowance was 180–300 and 3.0 m^2^/animal when at pasture and indoors, respectively.

Bulls were slaughtered at a mean age of 19.3 months. On the day of slaughter, the animals were transported (30 km), without the mixing of treatment groups, to a commercial abattoir and immediately slaughtered by bolt stunning followed by exsanguination from the jugular veins. Electrical stimulation was not applied, and carcasses were hung by the Achilles tendon. Post-slaughter, carcasses were weighed and graded for conformation (15-point scale, classes E+ (highest) to P− (lowest), E+ is 15) and fatness (15-point scale, scores 5+ (highest) to 1− (lowest), 5+ is 15) according to the EU Beef Carcass Classification Scheme [10]. Approximately 45 min after slaughter, carcasses were placed in a chill set at 8 °C. The pH and temperature decline of the LT at the 10th rib were recorded in the left side carcass [11]. At 1 h post-slaughter, a sample (ca. 30 g) of LT tissue (from the 9th rib position) was taken from the left side of the carcass, snap frozen in liquid nitrogen and maintained at −80 °C for metabolic enzyme activity and fibre typing analyses [12,13]. After approximately 10 h, the chill temperature was reduced to 0 °C.

At 48 h post-mortem, carcasses were cut at the 5/6th rib interface of the left side of the carcass, and muscle pH and temperature were recorded. The cut surface of the muscle was allowed to bloom for 90 min, and the colour (Hunter L, a, b) was measured using a Miniscan XE Plus (Hunter Associates Laboratory Inc., Reston, VA, USA). The L, a, b colour coordinates represent lightness (scale 0 (black) to 100 (white)), redness (scale +a (red) to –a (green)) and yellowness (scale +b (yellow) to −b (blue)) of the muscle, respectively. Chroma (saturation or colour intensity (C), computed as √(a^2^ + b^2^), where a higher ‘*C*’ value indicates higher colour saturation) and hue angle, H (computed as [tan^−1^(b/a)][180/π], where 0/360° is red, 90° is yellow, 180° is green and 270° is blue in colour) were also determined. The cube roll (commercial cut that begins between the 5th and 6th rib and ends between the 10th and 11th rib) was then removed. The LT from the cube roll was vacuum-packed, transported to Teagasc, Food Research Centre, Ashtown, Dublin and stored overnight at 2 °C. 

### 2.2. Colour and Chemical Analysis

The pH of the LT was measured after 72 h post-mortem, and the muscle was cut into individual steaks (thickness 25 mm). The steak closest to the 10th rib position was used for colour determination. Steaks were wrapped in oxygen-permeable polyvinylchloride film (oxygen permeability of 580 mL/m^2^ h at standard temperature and pressure) and allowed to bloom at 4 °C for 1 and 24 h. Colour as CIE L* (lightness), a* (redness) and b* (yellowness) was measured through the film at three locations on each muscle, using a dual beam spectrometer (UltraScan XE; Hunter Laboratories, Reston, VA, USA) and averaged. Hue angle (H*) and Chroma (C*) values were calculated as described above. Reflectance spectra from 400 to 700 nm at 10 nm intervals were also recorded. Reflectance data were used to estimate pigment proportions [14] and for the discriminant analysis below. Delta E (∆E), the variation in colour between 1 h and 24 h of aerobic display was calculated according to AMSA [15].

Another steak was vacuum-packed after cutting and stored at −20 °C prior to analysis of chemical composition [11] and total haem pigments [16]. Glycolytic enzyme activities (lactate dehydrogenase (LDH) and phosphofructokinase (PFK)) and oxidative enzyme activities (isocitrate dehydrogenase (ICDH), citrate synthase (CS) and cytochrome *c* oxidase (CCO)) were spectrophotometrically quantified [12]. Muscle fibre types were analysed using high-resolution mini-gel electrophoresis [13].

### 2.3. Statistical Analysis

For each carcass, the temperature of LT at pH 6.0 was estimated by interpolation of the linear relationship between temperature and the natural log of pH. Data were subjected to analysis of variance using Genstat (19th edition, VSN International, Hemel Hempstead, England) with a model that had treatment and block as main effects and animal as experimental unit. Data were adjusted, as appropriate, using ultimate pH or temperature of LT at pH 6.0 as a co-variate. Muscle colour data were also analysed according to a split-plot design with the above effects in the main plot and time and time-related interactions in the sub-plot. Multiple analysis of variance using SAS was used to calculate partial correlation coefficients (*p*), from the error sum of squares and cross products (SSCP) matrix, between selected carcass and muscle characteristics and colour variables of beef. All stated differences in subsequent sections were statistically significant at *p* < 0.05.

Discrimination models based on muscle colour data and visible reflectance spectra were built in R [17]. The potential of colour data (L*, a*, b*) to classify beef samples based on pre-slaughter diet was examined by canonical discriminant analysis (CDA) using the R packages: MASS [18] and Candisc [19]. Muscle reflectance spectra were transformed (log inverse of reflectance) and the potential of these data to classify beef samples based on pre-slaughter diet was examined using the partial least squares discriminant analysis (PLS-DA) algorithm included in the mixOmics R package [20]. Both CDA and PLS-DA models were validated using a 5-fold cross-validation with 50 repeats and evaluated by means of the total accuracy (i.e., percentage of samples correctly classified) and class error rates (i.e., percentage of samples misclassified by class). Loading values were used to assess how the different colour parameters or wavelengths contributed to the discrimination. For PLS-DA models, the variable importance in projection (VIP) score was also used to identify the most important wavelengths for the classification of beef according to cattle pre-slaughter diet. Three data combination/classification approaches were evaluated: (i) discrimination between G0, G0AL and AL; (ii) discrimination between G0 and AL; (iii) discrimination between G0 and C, where C was G0AL and AL samples combined.

## 3. Results

Unless otherwise indicated, all stated differences were significant (*p* < 0.05). 

### 3.1. Animal Growth and Carcass Characteristics

The average daily rainfall, daily maximum temperature and daily minimum temperature for the first 100 days of the study were 1.67 mm, 15.8 °C and 7.3 °C, respectively. The corresponding values for the second 100 days were 2.18 mm, 18.7 °C and 9.9 °C. Animal growth and carcass characteristics are shown in Table 1. Overall growth rate was lower for G0 animals than G0AL animals which, in turn, was lower than AL animals. G0 animals had lower pre-slaughter liveweight and carcass weight than G0AL animals, which had lower values than AL animals. Carcass fat classification was lower for G0 animals compared to G0AL and AL animals, which did not differ. The post-mortem temperature of muscle from G0 animals was lower at 1, 3, 5 and 7 h compared to muscle from G0AL animals which, in turn, was lower than muscle from AL animals (except for at 1 h) (Table 1). The post-mortem pH of muscle was similar between treatments at 1 h but at 3, 5 and 7 h was higher for G0 animals compared to muscle from G0AL and AL animals, which did not differ (Table 1). The temperature at which the pH of muscle reached 6.0 was lower for G0 animals compared to muscle from G0AL and AL animals, which did not differ (Table 1).

### 3.2. Muscle Ultimate pH and Colour

There was no difference in the pH of muscle at the fifth/sixth rib interface at 48 h post-mortem (Table 2). The colour of longissimus muscle at the fifth/sixth rib is shown in Table 2. Muscle from G0 animals was darker (lower L value) than muscle from G0AL and AL animals, which did not differ. The redness of muscle from G0 and G0AL animals was similar but less red (lower a value) than muscle from AL animals. Muscle from G0 animals was less yellow (lower b value) than muscle from G0AL and AL animals, which did not differ. The saturation of muscle from G0 and G0AL animals was similar but lower than muscle from AL animals. Adjustment for muscle pH did not change the above results (data not shown).

The pH measured at the 10th rib 72 h post-mortem was higher for muscle from G0 animals than muscle from G0AL and AL animals (Table 3). The colour of the longissimus muscle after 1 h aerobic display is shown in Table 3. There was no difference between treatment groups for lightness (L* value) or hue. The muscle from G0 animals was less red (lower a* value), less yellow (lower b* value) and less saturated than muscle from G0AL and AL animals, which did not differ. The metmyoglobin percentage in muscle from G0 and G0AL animals was similar but lower than muscle from AL animals. The deoxymyoglobin and oxymyoglobin percentages were lower in muscle from G0 than in muscle from G0AL and AL animals, which did not differ. Adjustment for muscle pH removed the differences in yellowness but did not affect the other colour variables. Adjustment for the temperature at which the longissimus muscle reached pH 6.0 removed all the differences between treatments, although there was a tendency (*p* = 0.07) for the difference in the metmyoglobin percentage to remain.

The colour of the longissimus muscle after 24 h aerobic display is shown in Table 4. There was no difference between treatment groups for lightness or yellowness. The muscle from G0 animals was less red and less saturated than muscle from G0AL and AL animals, which did not differ. The hue of muscle from G0 was higher than G0AL which, in turn, was higher than muscle from AL animals. The metmyoglobin percentage in muscle from G0 and G0AL animals was similar but lower than in muscle from AL animals, while the deoxymyoglobin and oxymyoglobin percentages did not differ between treatments. Adjustment for muscle pH tended (*p* = 0.06) to remove the differences in saturation but did not affect the other colour variables. When adjusted for the temperature at which the longissimus muscle reached pH 6.0, the metmyoglobin percentage was similar in muscle from G0AL and AL animals, but other variables were not affected. 

With respect to colour coordinates measured at the 10th rib, increasing aerobic exposure increased redness, yellowness, saturation and metmyoglobin and oxymyoglobin percentages and decreased the metmyoglobin percentage. As can be seen in a comparison of Table 3 and Table 4, there was an interaction between the finishing diet and the duration of aerobic display for deoxymyoglobin and oxymyoglobin percentages (there was no difference between treatments after 24 h of aerobic exposure) and for hue (there was no difference between treatments after 1 h of aerobic exposure). There was no difference between treatments for the change in overall muscle colour (Delta E) between 1 and 24 h of aerobic exposure, i.e., 6.86, 6.93 and 7.17 (sed 0.67) for G0, G0AL and AL, respectively.

### 3.3. Muscle Composition

The chemical composition of the longissimus muscle is shown in Table 5. Muscle from G0 animals had lower lipid and higher moisture concentration than muscle from G0AL animals which, in turn, was lower than muscle from AL animals. The activity of the glycolytic enzymes, LDK and PFK, and the oxidative enzyme, CCO, were lower in muscle from G0 animals than muscle from G0 and AL animals, which did not differ. There was no difference between production systems for the proportions of muscle fibre type IIA and IIX, but the proportion of type I tended (*p* = 0.09) to be lower in muscle from G0 animals than in muscle from AL animals. Muscle fibre type IIB was detected in three animals in G0 (representing 15.7 to 23.5% of muscle fibres in individual animals) and in three animals in G0AL (representing 20.2 to 29.7% of muscle fibres in individual animals) but not in animals in AL.

### 3.4. Correlations between Carcass or Muscle Characteristics and Muscle Colour

Correlations between carcass or muscle characteristics and colour parameters after 1 h of aerobic display are shown in Table 6. The L* value was positively correlated with LDH and PFK enzyme activity and with type IIX muscle fibres and negatively correlated with pH and with myoglobin concentration. The a* value was positively correlated with temperature at pH 6.0. The b* value was positively correlated with temperature at pH 6 and tended (*p* = 0.09) to be positively correlated with type IIX muscle fibres. The b* value was negatively correlated with pH, myoglobin concentration and CCO activity. The C* value was positively correlated with temperature at pH 6.0, negatively correlated with CCO activity and tended (*p* = 0.096) to be negatively correlated with pH. Hue angle was positively correlated with LDH and PFK activity and type IIX muscle fibres, negatively correlated with fat score and with myoglobin concentration and tended (*p* = 0.065) to be negatively correlated with type IIA muscle fibres.

Correlations between carcass or muscle characteristics and colour parameters after 24 h of aerobic display are also shown in Table 6. The L* value was positively correlated with LDH and PFK enzyme activity and with type IIX muscle fibres and negatively correlated with pH, myoglobin concentration, ICDH activity, type IIA muscle fibres and type I fibres (*p* = 0.056). The a* value was positively correlated with fat score, myoglobin concentration temperature at pH 6.0 and negatively correlated with type IIA muscle fibres (*p* = 0.053). The b* value was positively correlated with temperature at pH 6.0 and type IIX muscle fibres, was negatively correlated with type IIA muscle fibres and tended (*p* = 0.074) to be negatively correlated with pH. The C* value was positively correlated with conformation score, myoglobin concentration and temperature at pH 6.0 and negatively correlated with type IIA muscle fibres. Hue angle was positively correlated with LDH activity and type IIX muscle fibres and was negatively correlated with myoglobin concentration and ICDH activity. 

### 3.5. Classification of Beef According to Pre-Slaughter Cattle Diet

**Figure 1:**
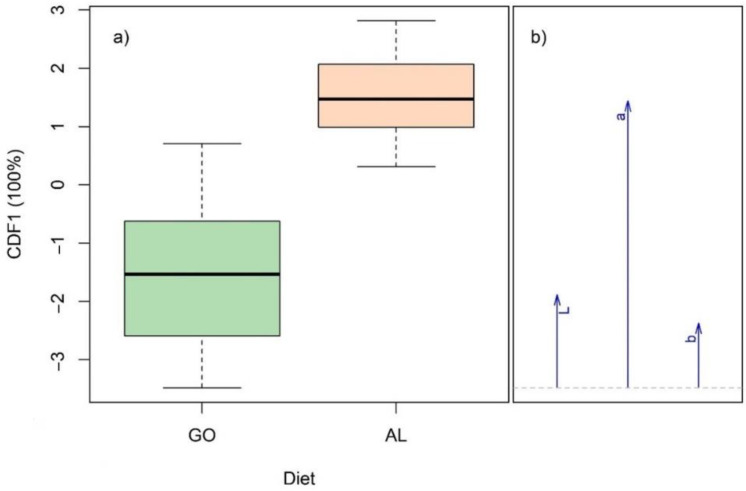
Canonical score (**a**) and structure coefficient (**b**) plots for the 1st canonical discriminant function (CDF1) of the model build to discriminate G0 from AL samples using L*, a* and b* data recorded after 24 h of air exposure.

When using L*, a* and b* data, the best classification was observed when using the data for LT exposed to air for 24 h and when only considering the more extreme pre-slaughter diets i.e., G0 and AL (Table 7). This model had a total accuracy of 90% and included one canonical discriminant function (Figure 1). The a* value was the main colour parameter for the discrimination of G0 from AL, where beef samples with high a* values were classified as AL, while samples with low a* values were classified as G0.

**Figure 2:**
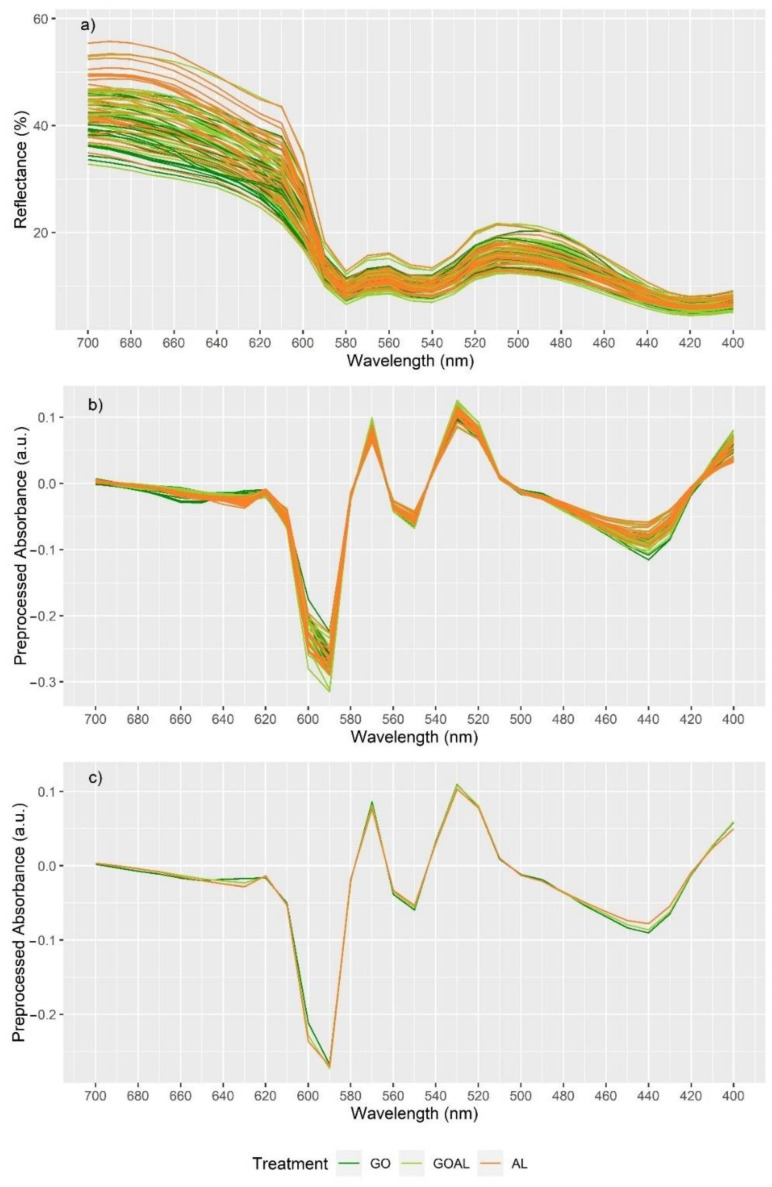
Visible reflectance spectroscopy of beef samples exposed to air for 24 h: (**a**) Individual reflectance spectra; (**b**) Individual absorbance spectra pre-processed using the Savitzky–Golay with 1^st^ derivative algorithm; (**c**) average pre-processed spectra for GO, GOAL and AL samples.

**Figure 3:**
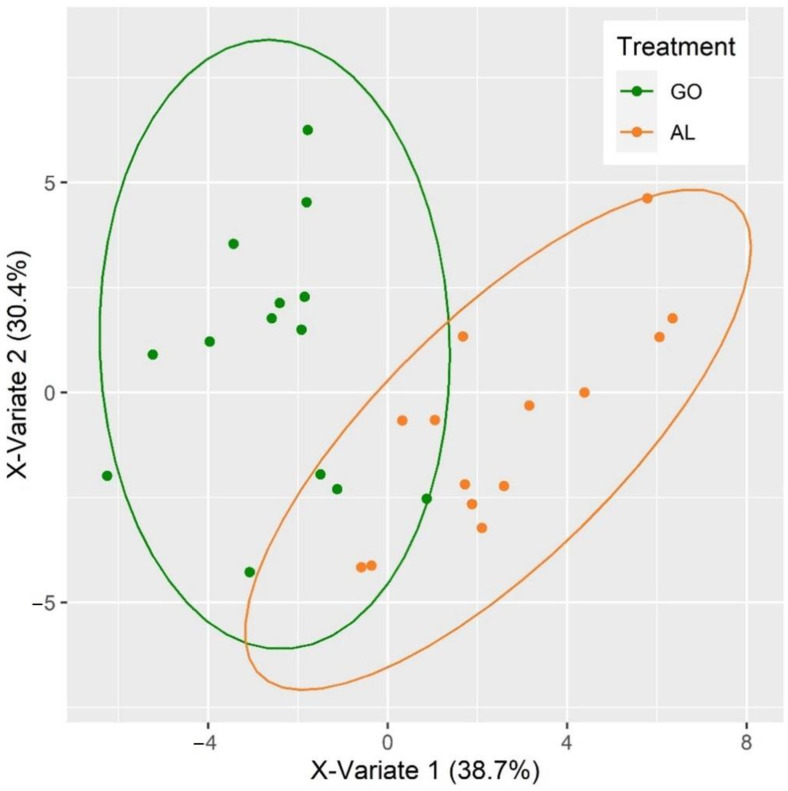
Score plot of the PLS-DA model for classification of G0 and AL samples build with spectra collected after exposure to air for 24 h and pre-processed using the Savitzky–Golay algorithm.

Similarly, when considering the reflectance spectrum (Figure 2a), lower class error rates were observed for samples exposed to air for 24 h and when only G0 and AL were considered (Table 8). Of the two spectral pre-processing techniques, Savitzky–Golay with 1st derivative (Figure 2b,c) provided slightly better results with a total model accuracy of 89%. Two was the optimal number of PLS-DA factors to be included in the model (i.e., lowest total error rate). The score plot obtained for this model (Figure 3) indicated that samples with low score values in PLS-DA 1 and high score values in PLS-DA 2 were identified as G0 samples, while the opposite was associated with AL samples. This relationship was further explored through the loading plots obtained for the model (Supplementary Figure S1). The loading plot of PLS-DA 1 (Supplementary Figure S1a) revealed that all signals associated with the AL dietary treatment (coloured in orange) had a positive loading value, while all signals associated with the G0 dietary treatment (coloured in green) had a negative loading value. In contrast, in PLS-DA 2 (Supplementary Figure S1b), positive loading values were mostly associated with G0 samples and negative loading values with AL samples. The relevance of each wavelength was assessed through the variable importance in projection (VIP) score (Supplementary Figure S1c). Variables/wavelengths with a VIP score higher than one are considered to be important [21]. The most important wavelengths (VIP > 1.1) for the classification of Go and AL samples were (in order) 640, 630, 690, 680, 560, 600, 460, 570, 470, 550 and 620 nm. Higher absorbance at 640, 630, 600 and 570 nm were associated with G0 samples, while higher absorbance at 690, 680, 560, 460, 470, 550 and 620 nm were associated with AL samples. 

## 4. Discussion

### 4.1. Animal Growth and Carcass Characteristics

The overall growth and carcass weight data reflected the energy consumption by the three groups. The higher growth rate of the G0AL bulls in the second part of the grazing season compared to AL bulls reflected compensatory growth in the G0AL group. All measured indices of fatness also reflected energy consumption and associated carcass weight. Carcasses from the G0 bulls did not, on average, achieve the carcass fatness classification requirement of 2+ (6 on a 15-point scale) set by the beef industry. Thus, while G0 bulls would comply with one definition of “grass-fed” (220 +/− 40 d, [22]) and be eligible for the growing market for such beef, at present, they would attract a price penalty based on the lower carcass fatness classification. Choosing the most appropriate production system is therefore a decision for the individual producer. However, if muscle colour is adversely affected by long-term grazing, the attractiveness of this system will be diminished. In the context of this study, the G0AL group was included to examine if there were residual effects on muscle colour, composition and the structure of grazing in the first half of the grazing season. The post-mortem decline in LT temperature measured at the 10th rib reflected the slower cooling as carcass weight increased from G0 to G0AL and from G0AL to AL.

### 4.2. Ultimate pH

The ultimate pH, i.e., at 48 h in the present study, is mainly determined by muscle glycogen content at slaughter, which, in turn, is influenced by pre-slaughter nutrition and the stress level of the animal before and at slaughter [23]. In the present study, the animals were transported to the abattoir in their farm groupings to avoid aggressive behaviour that could occur due to mixing unfamiliar animals, and they were slaughtered immediately upon arrival, thereby minimising pre-slaughter stress. In the commercial abattoir where the bulls were slaughtered, carcasses are quartered at the fifth rib interface 48 h post-mortem. The pH of the LT measured at that site did not differ between treatments and was within the normal range [24]. While the pH of the LT measured at the 10th rib site at 72 h post-mortem differed significantly, the absolute values were similar for both sites. 

### 4.3. Muscle Colour

In the abattoir, the colour of the LT is assessed visually at the quartering point. We measured colour in the abattoir at this position on the LT using a portable spectrocolorimeter and the Hunter L, a, b system. This was to allow comparison with previous experiments and with Moran at al. [4] where bulls that grazed for 78 days or were indoors and fed a high concentrate diet for 91 days were slaughtered at the same time. The lower L and the a value for G0 compared to AL in the present study is consistent with Moran et al. [4] who also observed a numerically lower b value for the grass-fed bulls compared to the concentrate-fed bulls. Using a similar protocol to the one used in the present study, Moran et al. [11] observed no difference in L and b values between G0AL and AL bulls, which is consistent with the present study. While the a value also did not significantly differ, it was numerically lower for G0AL (22.4) compared to AL (23.0) and consistent with the present study. Despite the lower L value for G0, no carcass was deemed “dark cutters” by abattoir personnel. For retail sale, the striploin is usually harvested posterior to the 10th rib. Therefore, we also sampled muscle at this point and measured pH and colour under more controlled laboratory conditions. In this case, we used a bench-top spectrocolorimeter and the CIE Lab system to allow comparison more broadly with the literature. When measured at the 10th rib site, at 72 h post-mortem, there was no difference in L* value after 1 h or 24 h of exposure to air. This suggestion of an interaction between lightness and site of measurement may be due to variation in colour along the longissimus thoracis et lumborum muscle as described in [25] and possibly, the different susceptibilities at different positions of the LT to changes in the pre-slaughter management of the bulls. While it is recognised that comparison of the two locations is confounded by the methodology used and the timing of measurement, simultaneous assessment of treatment effects on lightness at several locations/muscles merit consideration, particularly from a commercial perspective. Nevertheless, the differences with respect to a* and b* values were consistent for both measurement sites, and these are reflected in the values for saturation and hue.

The colour of meat on retail display has a critical influence on consumer purchasing decisions. Consumers presume bright red meat to be fresher and of higher quality, whereas pale, discoloured, or darker meat is perceived to be nearing spoilage or of poorer quality [1], and grass-fed beef is frequently observed to be darker than concentrate-fed beef [2,26]. However, Acciaro et al. [27] found no difference in the colour of LT from Sarda bulls finished at pasture or indoors on concentrates. This may reflect the shorter duration of treatment (61 days compared to 199 days in the present study or indeed 78 days in Moran et al. [4]), grazing management or the nature of the pasture. The effects of pasture finishing may influence the suitability of beef for markets with colour specifications; for example, within Europe, Mediterranean markets have a preference for carcasses with lighter-coloured muscle [28]. Grass-fed beef as produced and assessed in the present study may not meet this particular specification. The less red muscle in the G0 animals in the present study is consistent with the literature [4,29,30]. Holman et al. [1] concluded that a* value provides a useful prediction of the consumer acceptability of beef colour. Thus, samples with a* value equal to or greater than 14.5 were acceptable to the consumer with a 95% confidence interval. Based on this criterion, beef from G0 that was exposed to air for 1 h would be less acceptable than beef from the concentrate-fed animals. Consumer studies with “grass-fed” beef are needed to confirm this. Muscle was also exposed to air for 24 h to reflect the atmosphere during display in a retail case. The increase in all colour variables with an increase in the duration of aerobic display is consistent with Moran et al. [31]. Beef exposed in this way was acceptable to the consumer based on [1] above. This indicates that comparisons between production systems at one display/exposure time point with respect to consumer acceptability should be made with caution, but also that the time at which beef is offered to the consumer is important to maximise the value of grass-fed beef.

### 4.4. Possible Explanations for the Differences in Muscle Colour

Having detected differences in muscle colour, the second objective of this study was to explore possible explanations for these differences. Gagaoua et al. [32] examined the effects of mainly production and carcass characteristics on the colour of muscle within a database of 887 Charolais cattle which included 446 young bulls. They reported that an increase in carcass fat classification (but not carcass weight), increased a* [32]. Djimsa et al. [33] reported that heavier carcasses had a higher a* value compared to lighter carcasses when similarly managed post-mortem which is consistent with the present study. This may be related to the rate at which carcases cool post-mortem, as discussed below. In the current study, the darker colour of the grass-fed beef at the fifth rib interface does not appear to be due to high ultimate pH, which did not differ and was in the range considered normal, as discussed above. The general modest impact of adjusting for differences in pH on the colour of LT at the 10th rib interface supports this suggestion. However, the small negative partial correlation between pH and lightness of the LT after 1 and 24 h of display indicates that there is a subtle relationship between these variables, as reported in [32].

The activity in which grazing animals can engage has been suggested to influence muscle colour through an increase in myoglobin concentration and therefore, redness [2]. The fact that there was no difference in myoglobin concentration between treatments despite the difference in space available to the bulls does not support this hypothesis. However, the positive partial correlation supports the relationship between a* and myoglobin concentration, albeit this correlation was only present for LT after 24 h of aerobic display. The redox state rather than the concentration of myoglobin was more related to colour as indicated by the negative correlation for the metmyoglobin percentage and the positive correlation for the oxymyoglobin percentage and lightness (and yellowness) at both display times. The difference in ultimate pH likely contributed to the difference in the redox state of the LT at initial display, but structural changes due to differences in pH and temperature early post-mortem may also have affected the achromatic light scattering properties of the muscle [34,35]. Nevertheless, with prolonged exposure, the differences in the oxidation state of the muscle pigments had largely disappeared, indicating that the colour stability of the muscle had not been impaired by carcass management post-mortem.

With respect to muscle metabolism, it has been reported that high energy intake favours glycolytic muscle metabolism [35]. The higher activities of LDH and PFK in LT from AL and G0AL compared to G0 animals is consistent with this. The lack of difference in glycolytic enzyme activity between G0AL and AL indicates that the energy supply in the finishing phase removed any effects due to the consumption of the lower energy dense pasture by G0AL bulls in the first half of the grazing season. The higher glycolytic metabolism was reflected, albeit it was not statistically significant, in a higher proportion of the more glycolytic Type IIX muscle fibres. The positive correlation between the activities of both glycolytic enzymes and Type IIX muscle fibres with LT L* supports a role for the type of metabolism in muscle and beef colour. Pasture-fed steers have been reported to have a more oxidative metabolism than similar concentrate-fed steers [15,30]. Based on these reports, the lower CCO activity, supported by the tendency towards a lower proportion of Type I oxidative fibres, was not expected. Whether this observation is related to animal gender remains to be elucidated. Nevertheless, the change in the ratio of Type IIA to Type IIX muscle fibres (1.39 vs. 1.22 for G0 and AL) can be interpreted as a switch to more oxidative fibres [36]. In addition, the negative correlation between ICDH activity and Type I muscle fibres with LT L* after 24 h of aerobic display supports the conclusion of Apaoblaza et al. [30] that a shift towards oxidative metabolism in grazing cattle contributes to darker muscle. The fact that the type IIB muscle fibre was identified in only six animals in the present study might indicate that expression of Type IIB fibre is breed specific as it was reported to be identified commonly in Blonde d’Aquitaine, a French beef breed [37].

**Figure 4:**
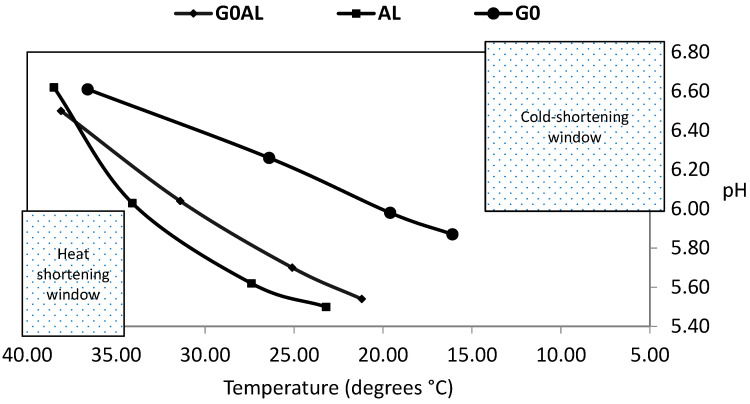
The post-mortem pH/temperature “window” in *Longissimus thoracis* from suckler bulls slaughtered after: 199 days at pasture (G0); 98 days at pasture followed by 101 days indoors and offered concentrates (G0AL); and 199 days indoors and offered concentrates (AL). Mean values per group.

Meat and Livestock Australia [38] consider the post-mortem pH-temperature “window’’ to be an important determinant of meat tenderness in particular. This window is shown in Figure 4, and it can be seen that none of the carcasses were likely to have been affected by ‘cold shortening’ (muscle pH > 6 at muscle temperature < 12 °C) or ‘heat shortening’ (muscle pH < 6 at muscle temperature > 35 °C). Rapid glycolysis/pH decline while temperature is high can result in a brighter and redder colour when muscle is first displayed [39]. This is consistent with the higher temperature at pH 6.0 and associated higher a* in LT from AL and G0AL animals compared to LT from G0 animals in the present study. In this regard, Hughes et al. [40] measured LT pH and temperature decline in 1512 beef cattle and colour at 14–31 h post-mortem using AUS-Meat colour chips. They reported that a high (40 °C) temperature at pH 6.0 generated the largest percentage (38%) of light meat colours (1B and 1 C on the AUS Meat system) and a low (15 °C) temperature at pH 6.0 generated the lowest percentage (10%) of light meat colours. They concluded that 25 °C was the optimum temperature for ensuring acceptable muscle colour. In the present experiment, the achieved temperature for G0AL and AL carcasses was close to this. Adjusting the chill settings to this target would be a practical strategy for abattoirs to minimise differences in muscle colour between lighter grass-fed and heavier concentrate-fed carcasses. The fact that virtually all colour differences were removed at both display times by using temperature at pH 6.0 as a co-variate in the statistical analysis should encourage this strategy. 

### 4.5. Discrimination of Pre-Slaughter Diet of Bulls

The identification of methods to authenticate the dietary history of cattle is important to allow the beef industry to protect brands such as “grass-fed”. When measuring muscle colour parameters (lightness, redness and yellowness) using a portable or bench-top spectrophotometer, the visible reflectance spectrum can be simultaneously obtained. If these data could also be used as a rapid authentication tool, if would obviate the need for more specialised and expensive spectroscopic equipment or slow and destructive chemical analysis. Few studies have examined the visible reflectance spectrum of beef in this regard.

In this study, discrimination models based on colour parameters (L*, a*, b*) and visible reflectance spectra had similar accuracy. The best classification models were obtained when only G0 and AL samples were considered. The decrease in classification accuracy when the G0AL category was included is likely due to the similarities between the G0AL and AL production systems—both systems involved finishing cattle indoors on ab libitum concentrates for 100 days prior to slaughter. This hypothesis was supported by the improvement in the accuracy of discrimination of G0 from G0AL and AL when the latter two groups were considered as one group. This outcome agrees with [8], who reported that the removal of the blended grain-fed category from the analyses of barley- and corn-fed beef, increased the percentage of correctly classified subcutaneous fat samples. The fact that the CDA model using the L*, a*, b* data identified the a* value as the main discriminating variable indicates that there is little additional value in this approach beyond the univariate analysis. In contrast, the fact that several wavelengths contributed to the discrimination in the PLS-DA model suggests that this approach could be advanced, in terms of a decrease in the wavelength interval, resulting in a more detailed spectrum and further chemometric analysis and therefore have applicability beyond the three dietary scenarios in the present study.

## 5. Conclusions

While meat from bulls finished at pasture was less red early post-mortem than meat from bulls finished on concentrates, the differences after 24 h aerobic display were small and unlikely to be of commercial importance. While long-term grazing caused subtle differences in muscle fibre distribution and metabolism, the major cause of the differences in redness was the relative post-mortem decline in pH and temperature. Adjusting the chill settings appears be a practical strategy for abattoirs to minimise early post-mortem differences in muscle colour between lighter grass-fed and heavier concentrate-fed carcasses. A combination of modified chill management and adequate retail display would ensure that consumer choice of bull beef would not be influenced by differences in colour. The preliminary results demonstrate the potential of both L*, a*, b* and visible reflectance spectra to discriminate between grass-finished and concentrate-finished bull beef. This could be a cost-effective strategy to protect “grass-fed” labels. However, further studies are required to determine if colour could be used to distinguish grass-fed beef from partially grass-fed beef (e.g., pasture supplemented with some concentrates) and from concentrate-fed beef. 

## Figures and Tables

**Table 1:** Growth, carcass characteristics, longissimus muscle pH and temperature decline post-mortem of suckler bulls slaughtered after: 199 days at pasture (G0); 98 days at pasture followed by 101 days indoors and offered concentrates (G0AL); and 199 days indoors and offered concentrates (AL).

Variable	G0	G0AL	AL	SED	Significance ^1^
Weight (kg)					
Initial	435	424	424	13.7	NS
Housing ^2^	545 ^a^	534 ^a^	616 ^b^	16.6	***
Final	639 ^a^	686 ^b^	741 ^c^	20.2	***
Age (days)	591	584	570	17.4	NS
Growth (kg/day)					
Turnout to housing	1.54 ^a^	1.43 ^a^	1.96 ^b^	0.092	***
Housing to slaughter	0.95 ^a^	1.50 ^b^	1.25 ^c^	0.114	***
Overall	1.18 ^a^	1.42 ^b^	1.60 ^c^	0.081	***
Carcass weight (kg)	359 ^a^	399 ^b^	436 ^c^	11.1	***
Fat classification ^3^	4.8 ^a^	7.5 ^b^	7.5 ^b^	0.44	***
Temperature (^o^C)					
Hours post-mortem					
1	36.6 ^a^	38.1 ^b^	38.5 ^b^	0.36	***
3	26.3 ^a^	31.4 ^b^	34.1 ^c^	0.86	***
5	19.5 ^a^	25.1 ^b^	27.4 ^c^	0.69	***
7	16.0 ^a^	21.2 ^b^	23.2 ^c^	0.71	***
pH					
Hours post-mortem					
1	6.61	6.50	6.62	0.053	NS
3	6.26 ^a^	6.04 ^b^	6.03 ^b^	0.072	**
5	5.98 ^a^	5.70 ^b^	5.62 ^b^	0.076	***
7	5.87 ^a^	5.54 ^b^	5.50 ^b^	0.058	***
Temperature (°C) @ pH 6.0	19.7 ^a^	29.7 ^b^	31.6 ^b^	1.28	***

^1^ ** = *p* < 0.01; *** = *p* < 0.001, NS = not significant. Within a row, means without or with a common superscript do not differ significantly. ^2^ Day on which G0AL bulls were moved indoors. ^3^ 15-point scale where 1 = leanest and 15 = fattest.

**Table 2:** pH and colour ^1^ of the longissimus muscle ^2^ of suckler bulls slaughtered after: 199 days at pasture (G0); 98 days at pasture followed by 101 days indoors and offered concentrates (G0AL); and 199 days indoors and offered concentrates (AL).

Variable	G0	G0AL	AL	SED	Significance ^1^
*5/6th rib interface* ^2^					
pH	5.61	5.59	5.57	0.025	NS
L ^3^	31.3 ^a^	34.5 ^b^	34.3 ^b^	1.10	*
a ^3^	20.3 ^a^	21.0 ^a^	22.5 ^b^	0.59	**
b ^3^	12.6 ^a^	13.4 ^b^	13.7 ^b^	0.36	*
Saturation	23.8 ^a^	24.9 ^a^	26.3 ^b^	0.64	**
Hue	31.8	32.7	31.4	0.62	NS

^1^ * = *p* < 0.05; ** = *p* < 0.01, NS = not significant. Within a row, means without or with a common superscript do not differ significantly. ^2^ Measured at 48 h post-mortem. ^3^ HunterLab where L, a, b = lightness, redness and yellowness, respectively.

**Table 3:** Colour ^1^ after 1-h aerobic display (beginning at 72 h post-mortem) of the longissimus muscle ^2^ of suckler bulls slaughtered after: 199 days at pasture (G0); 98 days at pasture followed by 101 days indoors and offered concentrates (G0AL); and 199 days indoors and offered concentrates (AL).

Variable	G0	G0AL	AL	SED	Significance ^2^
pH (72 h post-mortem)	5.61 ^a^	5.53 ^b^	5.51 ^b^	0.028	**
L*	45.3	45.5	45.8	0.79	NS
a*	12.5 ^a^	14.2 ^b^	15.2 ^b^	0.55	***
b*	10.6 ^a^	11.8 ^b^	12.2 ^b^	0.49	**
Saturation	16.4 ^a^	18.5 ^b^	19.5 ^b^	0.67	***
Hue	40.4	39.6	38.9	0.95	NS
% Metmyoglobin	20.8 ^a^	20.6 ^a^	22.1 ^b^	0.60	*
% Deoxymyoglobin	26.5 ^a^	18.6 ^b^	14.9 ^b^	2.53	***
% Oxymyoglobin	52.7 ^a^	60.8 ^b^	63.0 ^b^	2.33	***
L* ^3^	46.0	45.2	45.3	0.83	NS
a* ^3^	12.6 ^a^	14.2 ^b^	15.1 ^b^	0.63	**
b* ^3^	11.0	11.6	12.0	0.55	NS
Saturation ^3^	16.7 ^a^	18.4 ^b^	19.3 ^b^	0.76	*
Hue ^3^	41.1	39.4	38.5	1.05	NS
% Metmyoglobin ^3^	20.5 ^a^	20.7 ^a^	22.3 ^b^	0.68	*
% Deoxymyoglobin ^3^	26.0 ^a^	18.8 ^b^	15.2 ^b^	2.93	**
% Oxymyoglobin ^3^	53.5 ^a^	60.5 ^b^	62.5 ^b^	2.67	*
L* ^4^	45.5	45.4	45.6	1.37	NS
a* ^4^	13.2	13.9	14.7	0.92	NS
b* ^4^	11.2	11.5	11.9	0.84	NS
Saturation ^4^	17.4	18.1	18.9	1.12	NS
Hue ^4^	40.1	39.7	39.1	1.64	NS
% Metmyoglobin ^4^	20.4	20.8	22.3	1.04	0.069
% Deomyoglobin ^4^	24.6	19.3	16.1	4.36	NS
% Oxymyoglobin ^4^	55.0	59.9	61.6	3.99	NS

^1^ CIELab where L*, a*, b* = lightness, redness and yellowness, respectively. ^2^ * = *p* < 0.05; ** = *p* < 0.01, *** = *p* < 0.001. NS = not significant. Within a row, means without or with a common superscript do not differ significantly. ^3^ After adjustment for differences in pH. ^4^ After adjustment for differences in temperature @ pH 6.0.

**Table 4:** Colour ^1^ after 24-h aerobic display (beginning at 72 h post-mortem) of the longissimus muscle of suckler bulls slaughtered after: 199 days at pasture (GO); 98 days at pasture followed by 101 days indoors and offered concentrates (G0AL); and 199 days indoors and offered concentrates (AL).

Variable	G0	G0AL	AL	SED	Significance ^2^
L*	45.6	46.5	46.6	0.77	NS
a*	18.0 ^a^	19.7 ^b^	20.9 ^b^	0.62	***
b*	14.7	15.0	15.1	0.45	NS
Saturation	23.2 ^a^	24.8 ^b^	25.8 ^b^	0.71	**
Hue	39.3 ^a^	37.4 ^b^	35.9 ^c^	0.66	***
% Metmyoglobin	23.6 ^a^	23.4 ^a^	24.9 ^b^	0.48	**
% Deoxymyoglobin	6.1	5.8	5.1	0.57	NS
% Oxymyoglobin	70.3	70.8	70.0	0.85	NS
L* ^3^	46.4	46.2	46.1	0.79	NS
a* ^3^	18.0 ^a^	19.7 ^b^	20.8 ^b^	0.71	**
b* ^3^	15.0	14.9	14.9	0.50	NS
Saturation ^3^	23.4	24.7	25.6	0.81	0.061
Hue ^3^	39.7 ^a^	37.2 ^b^	35.6 ^c^	0.73	***
% Metmyoglobin ^3^	23.4 ^a^	23.4 ^a^	25.0 ^b^	0.55	**
% Deoxymyoglobin ^3^	5.6	6.0	5.5	0.60	NS
% Oxymyoglobin ^3^	71.0	70.6	69.5	0.92	NS
L* ^4^	46.0	46.3	46.3	1.33	NS
a* ^4^	19.5	19.2	19.9	0.96	NS
b* ^4^	15.6	14.7	14.5	0.73	NS
Saturation ^4^	24.9	24.2	24.7	1.11	NS
Hue ^4^	38.7	37.6	36.3	1.14	NS
% Metmyoglobin ^4^	23.0 ^a^	23.6 ^a,b^	25.2 ^b^	0.82	**
% Deomyoglobin ^4^	5.6	6.0	5.5	0.97	NS
% Oxymyoglobin ^4^	71.4	70.4	69.3	1.45	NS

^1^ CIELab where L*, a*, b* = lightness, redness and yellowness, respectively. ^2^ ** = *p* < 0.01, *** = *p* < 0.001. NS = not significant. Within a row, means without or with a common superscript do not differ significantly. ^3^ After adjustment for differences in pH. ^4^ After adjustment for differences in temperature @ pH 6.0.

**Table 5:** Characteristics of the longissimus muscle of suckler bulls slaughtered after: 199 days at pasture (G0); 98 days at pasture followed by 101 days indoors and offered concentrates (G0AL); and 199 days indoors and offered concentrates (AL).

Variable	G0	G0AL	AL	SED	Significance ^1^
Composition (g/kg)					
Lipid	7 ^a^	19 ^b^	28 ^c^	3.2	***
Moisture	756 ^a^	741 ^b^	734 ^c^	3.5	***
Protein	232	234	231	3.4	NS
Myoglobin	7.4	7.2	7.8	0.44	NS
Glycolytic enzyme activity ^2^					
LDH	839 ^a^	958 ^b^	974 ^b^	26.0	**
PFK	89 ^a^	148 ^b^	143 ^b^	9.0	***
Oxidative enzyme activity					
ICDH	0.86	0.80	0.81	0.056	NS
COX	9.2 ^a^	16.7 ^b^	17.0 ^b^	1.40	***
CS	4.8	4.6	4.6	0.48	NS
Fibre type profile (%)					
MyHC I	16.3	18.3	19.8	1.54	0.089
MyHC IIA	46.4	40.2	43.9	5.63	NS
MyHC IIX	33.4	36.6	35.9	6.47	NS

^1^ ** = *p* < 0.01, *** = *p* < 0.001. NS = not significant. Within a row, means without or with a common superscript do not differ significantly. ^2^ μmol/min/g of tissue; LDH = lactate dehydrogenase; PFK = phosphofructokinase; ICDH = isocitrate dehydrogenase; COX = cytochrome c oxidase; CS = citrate synthase.

**Table 6:** Correlations between carcass and muscle characteristics and muscle colour after adjustment for treatments effects.

		1-Hour Aerobic Exposure					24-Hour Aerobic Exposure				
		L*	a*	b*	C*	H*	L*	a*	b*	C*	H*
Carcass weight	r	−0.05	−0.03	−0.16	−0.10	−0.18	−0.03	0.13	0.03	0.10	−0.11
	P										
Fat score	r	−0.08	0.25	−0.05	0.13	−0.34	−0.12	0.38	0.26	0.36	−0.14
	P					0.031		0.014		0.019	
Conformation score	r	0.18	−0.09	0.06	−0.03	0.16	0.11	−0.03	−0.01	−0.03	−0.01
	P										
Intramuscular fat	r	−0.07	0.23	0.02	0.16	−0.23	−0.09	0.12	0.12	0.14	0.00
	P										
pH	r	−0.43	−0.17	−0.32	−0.26	−0.22	−0.35	−0.09	−0.28	−0.17	−0.20
	P	0.005		0.039			0.024		0.074		
Myoglobin	r	−0.39	0.15	−0.33	−0.06	−0.53	−0.37	0.56	0.18	0.47	−0.44
	P	0.012		0.037		0.000	0.017	0.000		0.002	0.004
%Met	r	−0.55	0.2	−0.42	−0.08	−0.75	−0.59	0.12	−0.53	−0.13	−0.73
	P	0.000		0.007		<0.0001	<0.0001		0.0003		<0.0001
%Deoxy	r	−0.09	−0.73	−0.48	−0.69	0.26	−0.61	−0.05	−0.49	−0.23	−0.49
	P		<0.0001	0.002	<0.0001		<0.0001		0.001		0.001
%Oxy	r	0.25	0.71	0.62	0.75	−0.06	0.74	−0.03	0.63	0.23	0.74
	P		<0.0001	<0.0001	<0.0001		<0.0001		<0.0001		<0.0001
Temperature at pH	r	0.21	0.39	0.30	0.39	−0.08	0.10	0.46	0.44	0.50	−0.07
	P		0.013	0.053	0.011			0.002	0.004	0.001	
ICDH	r	−0.22	0.08	−0.10	0.00	−0.18	−0.33	0.23	−0.15	0.09	−0.43
	P						0.034				0.005
LDH	r	0.40	−0.22	0.21	−0.03	0.50	0.37	−0.20	0.12	−0.09	0.37
	P	0.010				0.001	0.017				0.018
PFK	r	0.36	−0.17	0.16	−0.03	0.38	0.44	0.02	0.17	0.07	0.15
	P	0.021				0.013	0.004				
COX	r	−0.21	−0.23	−0.31	−0.30	−0.10	−0.11	−0.07	−0.22	−0.14	−0.17
	P			0.050	0.055						
CS	r	−0.02	−0.14	−0.05	−0.11	0.13	0.06	0.14	0.22	0.19	0.11
	P										
IIX	r	0.30	−0.04	0.27	0.10	0.40	0.45	0.11	0.40	0.23	0.32
	P	0.055				0.009	0.003		0.010		0.043
IIA	r	−0.24	−0.04	−0.25	−0.14	−0.29	−0.32	−0.31	−0.44	−0.38	−0.14
	P					0.065	0.041	0.053	0.004	0.013	
I	r	−0.08	−0.14	−0.20	−0.18	−0.10	−0.30	0.16	−0.02	0.11	−0.20
	P						0.056				

**Table 7:** Performances in 5-fold cross-validation with 50 repeats of the canonical discriminant analysis (CDA) models developed to discriminate beef according to animal production systems using colour variables (L*, a* and b*) after 1- or 24-h aerobic exposure.

Air Exposure	Classes	*n*	CDA Functions	Total Accuracy	Class Error Rates			
					G0	G0AL	AL	C
1 h	G0, AL	28	1	77%	24%	-	23%	-
24 h	G0, AL	28	1	90%	14%	-	6%	-
1 h	G0, G0AL, AL	43	1	48%	42%	70%	42%	-
24 h	G0, G0AL, AL	43	1	63%	26%	54%	31%	-
24 h	G0, C	43	1	83%	27%	-	-	12%

*n*: Total number of samples. G0: 199 days at pasture. G0AL: 98 days at pasture followed by 101 days indoors on concentrates. AL: 199 days indoors on concentrates. C: G0AL + AL samples combined.

**Table 8:** Performances in 5-fold cross-validation with 50 repeats of the PLS-DA models developed to discriminate beef according to animal production system using visible reflectance spectral data after 1- or 24-h aerobic exposure.

Air Exposure	Classes	*n*	Spectral Pre-Processing	PLS Factors	Total Accuracy	Class Error Rates			
						G0	G0AL	AL	C
1 h	G0, AL	28	Abs, SNV	2	78%	22%	-	21%	-
1 h	G0, AL	28	Abs, SG	1	84%	13%	-	14%	-
24 h	G0, AL	28	Abs, SNV	3	86%	19%	-	9%	-
24 h	G0, AL	28	Abs, SG	2	89%	18%	-	5%	-
1 h	G0, G0AL, AL	43	Abs, SNV	2	54%	34%	66%	37%	-
1 h	G0, G0AL, AL	43	Abs, SG	2	60%	24%	74%	20%	-
24 h	G0, G0AL, AL	43	Abs, SNV	3	64%	35%	52%	20%	-
24 h	G0, G0AL, AL	43	Abs, SG	5	60%	27%	70%	21%	-
24 h	G0, C	43	Abs, SG	2	81%	28%	-	-	15%

*n*: Total number of samples. G0: 199 days at pasture. G0AL: 98 days at pasture followed by 101 days indoors on concentrates. AL: 199 days indoors on concentrates. C: G0AL + AL samples combined. Abs: Absorbance. A = log (1/R). SNV: Standard Normal Variate. SG: Savitzky–Golay with 1st derivative, 3rd degree polynomial and smoothing window of 5 data points.

## Data Availability

The data presented in this study are available on request from the corresponding author.

## Supplementary Materials

The following supporting information can be downloaded at https://www.mdpi.com/article/10.3390/foods11152281/s1: Figure S1: Loading plots of the PLS-DA model for classification of G0 and AL samples built with spectra collected after exposure to air for 24 h and pre-processed using the Savitzky–Golay algorithm; (a) X-variate 1 and (b) X-variate 2. Colours indicate the dietary treatment in which the median is maximum for each wavelength. Figure 1c is the variable importance in projection (VIP) score for each wavelength.
