# Supplementary material for: Does Finishing at Pasture Influence the Colour of Muscle from Suckler Bulls and Can Colour Be Used to Authenticate Their Pre-Slaughter Diet?
Source: Foods. 2022 Jul 30;11(15):2281. doi: 10.3390/foods11152281 (PMC9368067; doi:10.3390/foods11152281)
Supplement: Supplementary file 1 [file foods-11-02281-s001.zip › foods-1805888-supplementary.pdf]

Does finishing at pasture influence the colour of muscle from suckler bulls and can colour be used to authenticate their pre-slaughter diet?

Aidan P. Moloney [1,*], Edward G. O'Riordan [1], Mark McGee [1], Brigitte Picard [2], Frank J. Monahan [3], Lara Moran [4,5] and Raquel Cama-Moncunill [3]

[1] Teagasc, Animal & Grassland Research and Innovation Centre, Grange, Dunsany, Co. Meath, C15PW93, Ireland; edward.oriordan@teagasc.ie (E.G.O.), mark.mcgee@teagasc.ie (M.M.);
[2] Institut National de Recherche pour l'Agriculture, l'Alimentation et l'Environnement (INRAE), VetAgro Sup, Unité Mixte de Recherche sur les Herbivores (UMR Herbivores), Université Clermont Auvergne, F-63122 Saint-Genès-Champanelle, France; brigitte.picard@inrae.fr
[3] School of Agriculture and Food Science, University College Dublin, Dublin, D04V1W8 Ireland; frank.monahan@ucd.ie
[4] Teagasc Food Research Centre, Ashtown, Dublin 15, Ireland; lara.moran@ehu.es
[5] Lactiker Research Group, Department of Pharmacy and Food Science, University of the Basque Country (UPV/EHU), 01006 Vitoria-Gasteiz, Spain
* Correspondence: aidan.moloney@teagasc.ie

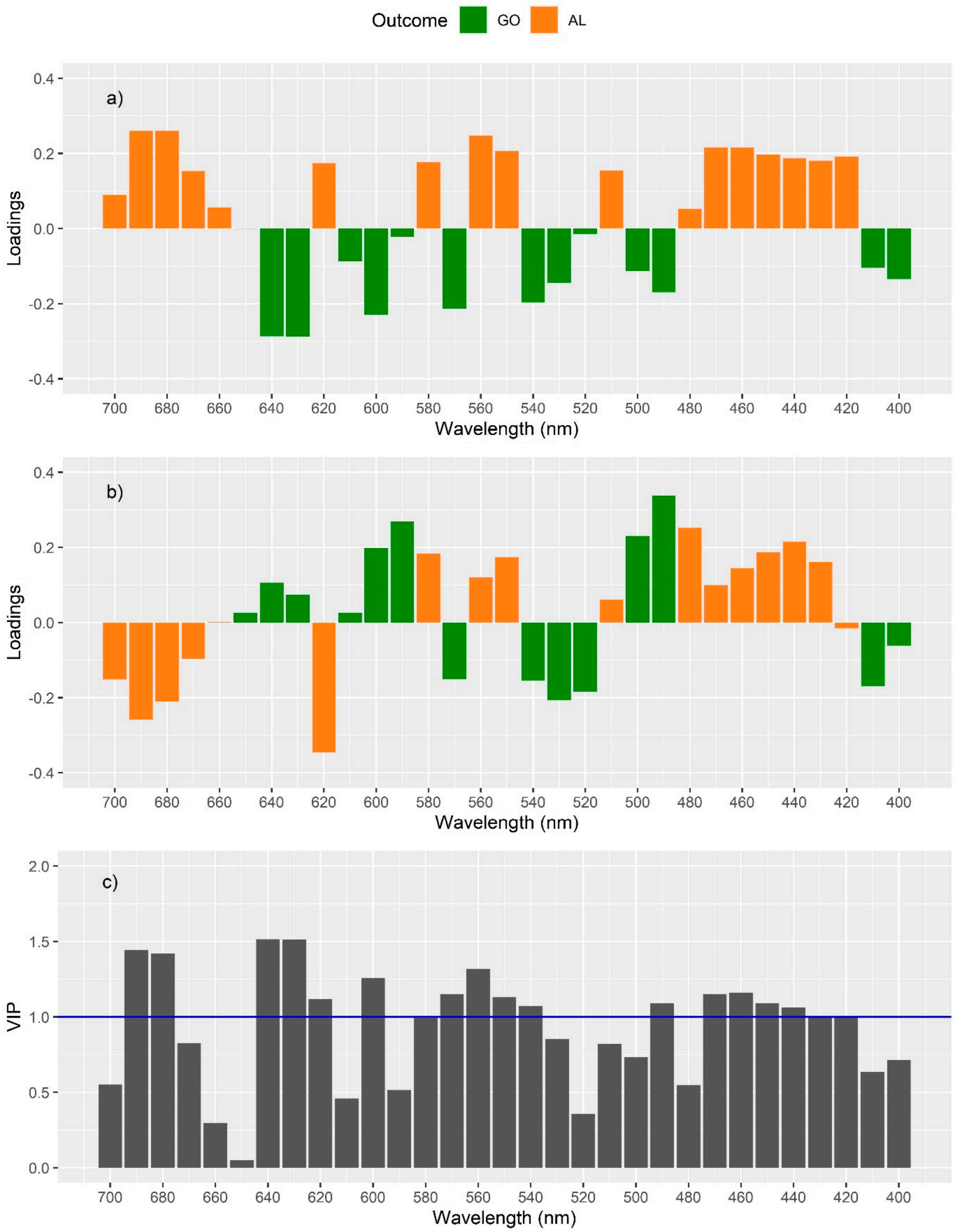

Supplementary Figure S1. Loading plots of the PLS-DA model for classification of G0 and AL samples built with spectra collected after exposure to air for 24h and pre-processed using the Savitzky-Golay algorithm: (a) X-variate 1 and (b) X-variate 2. Colours indicate the dietary treatment in which the median is maximum for each wavelength. Figure 1c is variable importance in projection (VIP) score for each wavelength.
